# Effects of ZnO Nanoparticles and Ethylenediamine‐*N*,*N*′‐Disuccinic Acid on Seed Germination of Four Different Plants

**DOI:** 10.1002/gch2.201800111

**Published:** 2019-06-26

**Authors:** Zeynep Görkem Doğaroğlu, Abdullah Eren, M. Fırat Baran

**Affiliations:** ^1^ Environmental Engineering Department Mersin University 33343 Mersin Turkey; ^2^ Vocational College of Kızıltepe Artuklu University 47200 Mardin Turkey; ^3^ Medical Laboratory Techniques Vocational Higher School of Healthcare Studies Artuklu University 47200 Mardin Turkey

**Keywords:** crop plants, nanoparticles toxicity, phytotoxicity, root elongation, wheat

## Abstract

The release of nanoparticles and biodegradable chelating agents into the environment may cause toxicological and ecotoxicological effects. The aim of this study is to determine the ecotoxic effects of zinc oxide (ZnO) nanoparticles and ethylenediamine disuccinic acid (EDDS) on most cultured four plants. The durum wheat, bread wheat, barley, and rye are exposed to 5 mL 10 mg L^−1^ ZnO nanoparticles and 10 mg L^−1^ EDDS in the seed germination stage. Results show that these different plant species have different responses to ZnO nanoparticles and EDDS. The germination percentage of bread wheat and rye decreases in the application of ZnO nanoparticles while the germination of durum wheat and barley increases as much as in radicle elongation and seedling vigor. While ZnO treatment causes a decrease in bread wheat and rye germinated rat in the range of 33–14.3%, respectively, there is no change in germination rate of these plants at EDDS treatment. In addition, EDDS treatment positively affects barley germination rate. In conclusion, it is clear that ZnO nanoparticles have more toxic effects on bread wheat and rye than EDDS, while barley is positively affected by ZnO nanoparticles and EDDS.

## 1 Introduction

The effects on the environment and humans of nanoparticles are not known well and they will be explicit a worldwide important environmental problem in the near future. Exposure to an accumulation of nanoparticles in aquatic, atmospheric, and terrestrial environmental compartments poses a risk to the environment and living organisms. The persistence of nanoparticles in the environment causes bioaccumulation in living organisms and affects the growth process.^1^ One of the most important parameters affecting growth is the enzyme system, and zinc (Zn) is one of the major element affecting the enzyme systems.^2^ Some nanoparticles such as gold (Au), silver (Ag), ZnO, and titanium dioxide (TiO_2_), at threshold concentrations, can contribute to the growth of plants by increasing nutrient uptake. Besides that, some organic materials such as ethylenediaminetetraacetic acid (EDTA) or ethylenediamine disuccinic acid (EDDS) can also be used as an auxiliary material to enhance plant growth.^3^ The presence of nanoparticles, at the above of threshold concentration in the soil‐plant system, can cause adverse effects in the environment. Determining the threshold concentrations of different nanoparticles is quite difficult because adverse effects differ depending on plants' species, nanoparticles' size and type. In our previous study, it has been determined that the ZnO and TiO_2_ nanoparticles, at about 30 nm mean size, did not show any significant effect on seed germination and root elongation of barley, on the other hand, antioxidative enzymes were adversely affected by these nanoparticles at different nanoparticles concentrations, especially from ZnO.^4^ By the way, it has been reported by many researchers that the ZnO nanoparticles variously effected on seed germination, seedling vigor, root elongation on wheat. These studies indicated the positive, negative, or ineffective impacts of ZnO nanoparticles depending on nanoparticles size and concentrations.^5^, ^6^, ^7^, ^8^ While Awasthi et al.^5^ and Solanki and Laura^8^ reported the positive effects of ZnO on wheat at about 13 nm (50 mg L^−1^ ZnO) and 40–50 nm (250 and 500 mg L^−1^ ZnO) mean size, respectively, on the other hand, Bagawade and Jagtap^7^ noted that ZnO nanoparticles (3–5 nm mean size) negatively affected wheat at the concentration of 1000–2000 mg L^−1^ ZnO. Due to these complicated data, it is not enough not only different nanoparticles types and size but also different plants type should be evaluated to determine the effects of nanoparticles in ecosystems. The researchers are able to ignore the effects of some organic acids when studying the effects of nanoparticles or trying to increase the phytoremediation capacity. Up to date, it has been believed that EDDS is cleaner than EDTA, but recently researchers noticed that it is not as innocent as it is believed. Fassler et al. observed EDDS promoted plant growth because of the decreased metal uptake and thus reduced heavy metal toxicity.^9^ On the other hand, other researchers indicated that plants (corn and bean) exposed to EDDS more inhibited than exposed to EDTA,^10^ or did not observe any growth inhibition in sunflower plants exposed to EDDS or EDTA.^11^

Determining the toxic effects of chemicals (e.g., nanoparticles, heavy metals, organic pollutants) can be done via different paths. Seed germination rate and root elongation tests are the most used phytotoxicity parameters. These parameters have several advantages as they need only seeds that can be obtained easily and the other requirements are inexpensive and sensitive processes,^1^, ^12^, ^13^ and they are suitable for all organic and/or inorganic pollutants. Root elongation, is an indicator test to determine the phytotoxic effects of test chemicals, was first shown by Ratsch and Johndro. The authors concluded that six different test chemicals on lettuce root elongation are effective for the chemicals tested.^13^, ^14^ After Ratsch and Johndro's study, some researchers started to investigate phytotoxic effects of different chemicals (organic or inorganic) using seed germination rate and root–shoot elongation of plants^15^ and the others investigated effects of different nanoparticles in the environment.^4^, ^6^, ^7^, ^8^, ^9^, ^10^, ^11^, ^12^, ^13^, ^14^, ^15^, ^16^, ^17^

Thus it was assessed and evaluated in this study that the toxic effects of ZnO nanoparticles and EDDS in four different plants (barley, bread wheat, durum wheat, and rye). In doing so, it was taken into consideration the seed germination rate, radicle–plumule elongation, and seedling vigor index of plants.

## 2 Results

### 2.1 Seed Germination

Seed germination is defined at different types depending on root elongation by different researchers. While Kordan^18^ described the seed germination as emerged rootlet or radicle before the shoot, Munzuroglu and Geckil^19^ described it as emerged 1 mm radicle, and Ren et al. (1996)^20^ described it as cracking of the seed coat. Meanwhile, Lin and Xing^17^ mentioned the seed germination as emergence radicle or cotyledon coming out from the seed coat. In this study, germination was accepted when the radicle or plumule came out of the seed coat.

**Figure 1:**
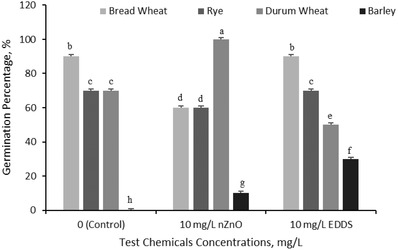
Germination percentage of bread wheat, rye, durum wheat, and barley seeds. Letter(s) on each bar show significant level (*p* < 0.01).

Four different plant seeds' germination, exposed to ZnO nanoparticles and EDDS, was determined after 7 d of treatment. While durum wheat and barley seeds were positively affected by ZnO nanoparticles treatment (*p* ≤ 0.01), bread wheat and rye seeds' germination was inhibited (*p* ≤ 0.01). The seeds of bread wheat and rye were almost never affected, and barley was positively affected by EDDS. On the other hand, the durum wheat seeds germination process was hindered by EDDS. Among the four plant seeds' species, the barley seeds were the most affected plant from ZnO nanoparticles and EDDS, as shown in **Figure** **1**. The maximum germination percentage was determined at 10 mg L^−1^ ZnO nanoparticles treatment on durum wheat, as 100% and the minimum germination was found at control in barley. Barley seeds treated with pure water did not germinate, while ZnO nanoparticles and EDDS treatment accelerated the germination process for barley (*p* ≤ 0.01).

### 2.2 Radicle–Plumule Elongation and Seedling Vigor

**Figure 2:**
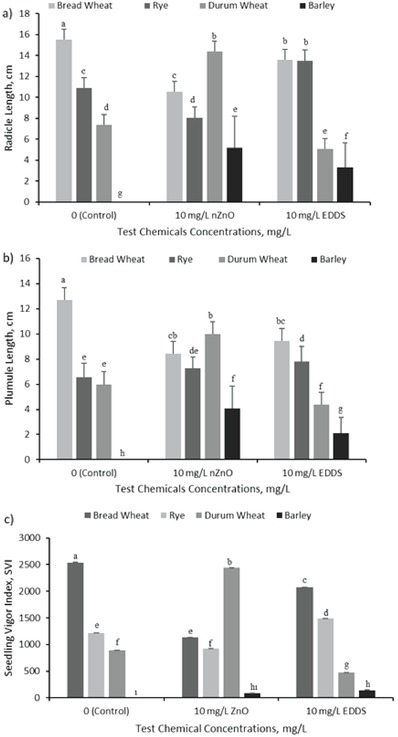
a) Radicle and b) plumule length and c) seedling vigor of four different plants. Letter(s) on each bar show significance level (*p* < 0.01).

Radicle and plumule elongation of germinated plants were determined using millimetric paper after germination time. As shown in **Figure** **2**a, the maximum and minimum radicle length was determined in bread wheat (as 15.52 cm) at control and in barley (as 1.73 cm) at 10 mg L^−1^ ZnO nanoparticles treatment, respectively. ZnO nanoparticles and EDDS treatments negatively affected radicle elongation of bread wheat with respect to control (*p* ≤ 0.01), and the minimum radicle length of bread wheat were determined at 10 mg L^−1^ ZnO nanoparticles treatment, as 10.20 cm. Radicle elongation of rye enhanced with EDDS treatment and decelerated with ZnO nanoparticles treatment (*p* ≤ 0.01). ZnO nanoparticles treatment was the most effective application for durum wheat between four plants seedlings. The maximum and minimum radicle length of durum wheat was measured as 14.63 and as 4.75 cm, respectively. EDDS and ZnO nanoparticles treatments accelerated the germination and seedling growth of barley (*p* ≤ 0.01). Any germination and radicle–plumule formation were not observed in barley at control. 3.31 cm radicle length was measured at 10 mg L^−1^ EDDS treatment, and 5.20 cm radicle length of barley was measured at 10 mg L^−1^ ZnO nanoparticles treatment.

The maximum and the minimum plumule length were determined in bread wheat at control and in barley at 10 mg L^−1^ EDDS treatment as 12.70 and 2.1 cm, respectively (Figure 2b). Durum wheat and barley, exposed to test chemicals, seedlings plumule elongation showed a similar trend, while bread wheat seedlings' plumule elongation showed the opposite trend to them. ZnO nanoparticles and EDDS treatments enhanced the plumule elongation in rye and barley (*p* ≤ 0.01) compared to control. The maximum plumule length of rye was observed at 10 mg L^−1^ EDDS treatment as 8.07 cm and on the other hand, 7.43 cm plumule length of rye, as the minimum length of rye, was measured at ZnO nanoparticles treatment. These lengths for barley were 2.13 cm at EDDS treatment and 4.08 cm at ZnO nanoparticles treatment (*p* ≤ 0.01). EDDS treatment was more effective than ZnO nanoparticles in plumule elongation of bread wheat. Besides that, both of these treatments inhibited the plumule elongation compared to control (*p* ≤ 0.01). This tendency was the opposite of the durum wheat. ZnO nanoparticles treatment was more effective than EDDS treatment in durum wheat. 9.98 cm, the maximum plumule length of durum wheat, was measured at ZnO nanoparticles treatment.

Seedling vigor indexes (SVI) of plants depends on their germination percentage and radicle–plumule length. The maximum SVI was calculated in bread wheat in control group as 2540, and the minimum SVI was calculated in barley at ZnO nanoparticles treatment as 92.70. Calculated SVI values of bread wheat and rye showed an almost similar trend. Seedling vigor was 2540, 1136.67, and 2073 at different treatment (control, ZnO nanoparticles, and EDDS, respectively) for bread wheat and 1221.89, 929.13, and 1510.44 SVI values were calculated respectively for rye. On the other hand, for durum wheat, 936.44 SVI was calculated at control, and about two and a half‐fold SVI was calculated at ZnO nanoparticles treatment as 2436.67. The minimum SVI was calculated for durum wheat at EDDS treatment as 471.67. The minimum SVI of barley was calculated at ZnO nanoparticles treatment as 92.70 and 163.23 SVI value was calculated at EDDS treatment (Figure 2c).

## 3 Discussion and Conclusion

Nanomaterials have a size of at least one dimension below 100 nm.^17^, ^21^ In many areas, their increasing use, especially in agricultural products, has created a sensation on different researchers. According to Sturikova et al.,^22^ nanofertilizers are good option to obtain more yields from agricultural plants. If they cause increasing the seed, yield, and biomass without toxic effects, the nanofertilizers can be conveniently used. For this reason, there is an increasing amount of research on the nanotoxicology. The release of nanomaterials in the environment causes the increasing exposure probability of plants to nonnatural nanomaterials, although plants have adapted to the presence of natural nanoparticles.^21^ Studies on the interaction between nanoparticles and plants and the interaction mechanisms are still in their early stages.^22^, ^23^ According to US EPA guidelines, if the nanoparticles have no adverse effects on plants, it can be reported that the minimal toxicity was occurred.^24^ The particle solubility is one of the most important factors on the bioavailability, toxicity, and fate, and it is as important as concentration and size. ZnO is classified as insoluble, even though ZnO metal oxides are appreciably soluble.^22^, ^25^, ^26^ Franklin et al.^25^ and Du et al.^26^ noted that ZnO nanoparticles' toxic effects occur due to dissolved ions; while on the other hand, Lin and Xing^27^ underlined that phytotoxic effects cannot be explained via dissolved ZnO nanoparticles. The aggregation, observed in this study, is a normal property of ZnO nanoparticles and the ZnO solution was stirred continuously that try to hinder aggregation. Actually, the main concern of nanoparticles toxicity is how it changes the effects of small size on organisms. These smaller particles have more mobility, more bioavailability, and more uptake by organisms than bulk particles size.^25^ The most studied ZnO nanoparticles size is <50 nm^5^, ^7^, ^27^, ^28^ and it was chosen 30 nm ZnO nanoparticles in this study. If ZnO nanoparticles size is smaller than the plant cell pores, nanoparticles can be absorbed and internalize into the plant cells. This interaction may affect plant growth from seed germination to yield production.^29^ Seed germination process starts with water imbibition and continued with the emergence of the radicle. The seed coat has a semipermeable structure and it protects the embryo from pollutants. Pollutants may not any effects on seed germination even though they have adverse effects on root elongation.^17^ The reason for this result was about firstly the structure of seed coat and then the smaller particles can easily penetrate to roots. By the way, Sturikova et al.^22^ and Garcia‐Gomez et al.^30^ mentioned that the effects of ZnO nanoparticles varied depending on plants species. This may explain that seed germination, radicle and plumule elongation of four different plants in this study was varied by nanoparticles depending on plants species. In this study, at the concentration of 10 mg L^−1^ ZnO nanoparticles concentration had an inhibitory effect on bread wheat (*Triticum aestivum* L. İkizce) and provocative effect on barley (*Hordeum vulgare* L. Ramata). These contradictory effects on rape, ryegrass, Zea mays,^17^ maize,^31^, ^32^ and wheat and barley^4^, ^33^ were reported in the literature, before.

The other factors to determine the toxic effects of nanoparticles are concentration and size. It was reported that if the exposure concentration of nanoparticles is low, there may not be able to observe toxic effects (but not always) on seed germination and/or radicle–plumule elongation.^5^, ^22^, ^27^, ^29^ However, there is some opposite effect in the literature. For example, Afrayeem and Chaurasia^34^ reported that the seed germination, root, and shoot elongation of chili reached the maximum value at high ZnO nanoparticles while decreasing was observed at low concentration. In this study, it was evaluated the ZnO nanoparticles at 10 mg L^−1^ concentration and in the size of 30 nm and classified as low concentration according to literature. In the results, the inhibition was determined in bread wheat and rye, while the opposite effects were determined in durum wheat and barley. In the nature of plants, Zn is a required micronutrient to healthy growth at low concentrations and also it cannot be found at high concentration in the environment both intentionally and accidentally. So the results of high concentrations cannot be representative of real life. Thus it was chosen a low concentration in this study.

The other pollutant used in this study was EDDS, known as a biodegradable organic chelator synthesizing by some microorganisms naturally. It has been known to be harmless on organisms up to now, but it has now been revealed that it has some negative effects. EDDS was used in many studies^35^, ^36^, ^37^ as an assistant in phytoremediation processes, such as enhanced phytoremediation capacity. The other investigation, conducted by Doğaroğlu and Köleli,^33^ aimed to determine if EDDS and ZnO nanoparticles enhanced the phytotoxic effects of TiO_2_Ag nanoparticles on rye, or not. The results showed that ZnO nanoparticles and EDDS decreased the negative effects of only TiO_2_Ag nanoparticles treatment.^33^ In this study, it was determined whether the plants exposed to EDDS in the early stage of growth (in the germination process) affected or not. In the results, the response of seeds to EDDS varied depending on plants species. The germination percentage of barley positively affected in comparison with control, while bread wheat and rye were not significantly affected and also the germination percentage of durum wheat was negatively affected from EDDS.

Consequently, it should be evaluated that the effects of different chemicals on the environment. Even the chemicals we know as harmless (e.g., EDDS and ZnO nanoparticles) can have negative effects on the living organisms, especially on the plants that form the basis of the food chain. But when the researchers investigate the effects of chemicals, they should be very careful and try to not disturb to ecosystems.

## 4 Experimental Section

**Figure 3:**
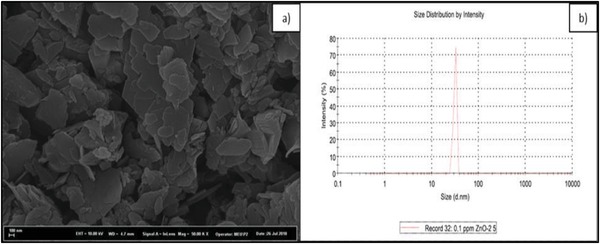
a) SEM image and b) size measurement of ZnO nanoparticles using zeta‐sizer.

*Test Materials*: Because of their large surface area, nanoparticles are more reactive than their bulk forms in biological systems. Zinc oxide nanoparticles were synthesized using a combination of sol–gel and hydrothermal methods with a small change as described by Doğaroğlu and Köleli^6^ and Ito et al.^38^ Zinc oxide nanoparticles' size was determined using zeta‐sizer and it was found 31.54 nm as shown in **Figure** **3**. It is clear that the ZnO nanoparticles have amorphous and flaky particles shapes.

Ethylenediamine disuccinic acid (EDDS) was prepared at the concentration of 10 mg L^−1^ from commercial EDDS (≈35% in H_2_O, Sigma‐Aldrich). The average size of ZnO nanoparticles was determined using zeta‐sizer Ver. 6.32 (Malvern Instruments Ltd.) using a 1 mL disposable cuvette. 10 mg L^−1^ ZnO nanoparticles and 10 mg L^−1^ EDDS solutions were prepared from 100 mg L^−1^ stock ZnO nanoparticles solution and from 40 × 10^−3^ m EDDS stock solution, respectively. Four different cereal crop seeds were used in this study: Bread wheat, barley, durum wheat, and rye plants. These seeds were purchased from Mersin Province, Turkey.

*Treatments and Measurements*: Laboratory studies provide more uniform conditions than field studies. Therefore, experiments were performed using ten seeds in 10 cm Petri‐dishes under laboratory conditions. The selected uniform seeds were selected to minimize error in germination tests. The sterilization process of seeds was conducted as given by Doğaroğlu and Köleli.^4^, ^6^ Double‐layer of filter paper cut and placed in Petri dishes. Filter paper in Petri dishes is generally used in the germination experiments as inert material.^7^, ^8^, ^39^ Ten seeds were placed in Petri‐dishes and were separately treated with 5 mL 10 mg L^−1^ ZnO nanoparticles and 10 mg L^−1^ EDDS test solutions. Control groups were treated with pure water and all treatments were conducted in three replications. Petri‐dishes placed in a dark chamber for 7 d at 25 °C. Thereafter, the number of germinated seeds was recorded on the seventh day and on average five plants in every petri‐dish were chosen to determine radicle and plumule length. Germination percentage (%) was calculated as described by Mahmoodzadeh et al.^40^ in the Equation (1) and SVI was calculated by Prasad et al.^41^ in the Equation (2)

(1) $$\mathrm{Germination}\;\mathrm{percentage}\;\;\left(\mathrm{GP}\right)\;\;=\;\;\mathrm{TNSG/TNST}\;\;\times \;\;100$$

Where; TNSG is the total number of seeds germinated; TNST is the total number of seed tested.

(2) $$\mathrm{Seed}\;\mathrm{Vigor}\;\mathrm{Index}\;\;=\;\;\mathrm{GP}\left(\%\right)\;\;\times \;\;\left(\mathrm{Root}\;\mathrm{length}\;\;+\;\;\mathrm{Shoot}\;\mathrm{length}\right)$$

*Statistical Analysis*: The statistical significance levels of difference for all measurements were evaluated using SPSS Version 22.0 software (SPSS, USA) at the level of *p* ≤ 0.01 with one‐way analysis of variance (ANOVA).

## Conflict of Interest

The authors declare no conflict of interest.
